# ECCB 2012 Organization

**DOI:** 10.1093/bioinformatics/bts418

**Published:** 2012-09-03

## CONFERENCE CHAIR

Torsten Schwede, Biozentrum Universität Basel & SIB Swiss Institute of Bioinformatics, Basel, Switzerland

## CONFERENCE CO-CHAIR

Dagmar Iber, ETHZ & SIB Swiss Institute of Bioinformatics, Basel, Switzerland

## LOCAL ORGANIZING COMMITTEE

Torsten Schwede, Biozentrum Universität Basel & SIB Swiss Institute of Bioinformatics, Basel, Switzerland

Katja Jenni, Biozentrum Universität Basel, Switzerland

Rita Manohar, Biozentrum Universität Basel, Switzerland

Sarah Güthe, Biozentrum Universität Basel & SIB Swiss Institute of Bioinformatics, Basel, Switzerland

Yvonne Steger, Biozentrum Universität Basel, Switzerland

Lorenza Bordoli, Biozentrum Universität Basel & SIB Swiss Institute of Bioinformatics, Basel, Switzerland

Irene Perovsek, SIB Swiss Institute of Bioinformatics, Lausanne, Switzerland

Jocelyne Bocquet, SIB Swiss Institute of Bioinformatics, Lausanne, Switzerland

Melissa Davis, SIB Swiss Institute of Bioinformatics, Lausanne, Switzerland

Ron Appel, SIB Swiss Institute of Bioinformatics, Lausanne, Switzerland

## STEERING COMMITTEE

Anna Tramontano (chair), University of Rome ‘La Sapienza’, Italy

Janet Thornton, EBI European Bioinformatics Institute, UK

Michal Linial, Hebrew University, Jerusalem, Israel

Ron Appel, SIB Swiss Institute of Bioinformatics, Switzerland

Alfonso Valencia, Centro Nacional de Investigaciones

Oncologicas, Madrid, Spain

Yves Moreau, University of Leuven, Belgium

Martin Vingron, Max Planck Institute for Molecular Genetics, Berlin, Germany

## PROGRAMME COMMITTEE—AREA CHAIRS

### A. Applied and Translational Bioinformatics

Julio Saez-Rodriguez, European Bioinformatics Institute (EMBL-EBI)

Ioannis Xenarios, University of Lausanne & SIB, Switzerland

### B. Bioimaging, Spatial-temporal Modeling, and Data Visualization

Sean O'Donoghue, CSIRO & Garvan Inst., Australia

Dagmar Iber, ETHZ & SIB, Basel, Switzerland

### C. Bioinformatics of Health and Disease, Biomarkers, and Personalized Medicine

Yves Moreau, University of Leuven, Belgium

Niko Beerenwinkel, SIB and ETHZ, Basel, Switzerland

### D. Databases, Ontologies, and Text Mining

Alfonso Valencia, Centro Nacional de Investigaciones Oncologicas, Madrid, Spain

Dietrich Rebholz-Schuhmann, European Bioinformatics Institute (EMBL-EBI)

### E. Evolution, Phylogeny, and Comparative Genomics

Nicolas Galtier, CNRS, Université Montpellier 2, France

Marc Robinson-Rechavi, University of Lausanne & SIB, Switzerland

### F. Macromolecular Structure, Dynamics, and Function

Anna Tramontano, University of Rome ‘La Sapienza’, Italy

Torsten Schwede, SIB & Biozentrum, University of Basel, Switzerland

### G. Mutations, Variations, and Population Genomics

Sven Bergmann, University of Lausanne & SIB, Switzerland

Richard Durbin, Wellcome Trust Sanger Institute, Hinxton, UK

### H. Protein Interactions, Molecular Networks, and Proteomics

Rob Russel, University of Heidelberg, Germany

Christian von Mering, University of Zurich & SIB, Switzerland

### I. Regulation, Pathways, and Systems Biology

Thomas Höfer, German Cancer Research Center (DKFZ) Heidelberg, Germany

Fabian Theis, Helmholtz Zentrum München, Germany

### J. Sequencing and Sequence Analysis

Martin Vingron, Max Planck Institute for Molecular Genetics, Berlin, Germany

Gaston Gonnet, ETH Zürich & SIB, Switzerland

### K. Technology and Software (TechTrack)

Jacques Rougemont, EPFL, Lausanne, Switzerland

Gert Vriend, Centre for Molecular and Biomolecular Informatics, Nijmegen, The Netherlands

### L. Workshops and Tutorials

Frederique Lisacek, SIB and University of Geneva, Switzerland

### M. Poster Chair

Michael Stadler, Poster chair, SIB & FMI Friedrich Miescher Institut, Basel, Switzerland

## PROGRAMME COMMITTEE MEMBERS

Mohamed Abouelhoda

Jan Aerts

Pankaj Agarwal

Christian Ahrens

Mario Albrecht

Patrick Aloy

Gregoire Altan-Bonnet

Andre Altmann

Inka Appel

Kazuharu Arakawa

Saul Ares

Susanna Assunto-Sansone

Philip Awadalla

Francisco Azuaje

Rolf Backofen

Ruth Baker

Pedro Ballester

Matteo Barberis

Geoff Barton

Thomas Bataillon

Alex Bateman

Michael Beckstette

Niko Beerenwinkel

Tim Beißbarth

Asa Ben-Hur

Nir Ben-Tal

Andreas Bender

Sven Bergmann

Andreas Bergner

Simon Berneche

Conrad Bessant

Joanna Betts

Inanc Birol

Judith Blake

Nils Blüthgen

Brigitte Boeckmann

Danail Bonchev

Paola Bonizzoni

Lorenza Bordoli

Erich Bornberg-Bauer

Anne-Laure Boulesteix

Phil Bourne

Yana Bromberg

David Bryant

Javier Buceta Fernández

Janusz Bujnicki

Anita Burgun-Parenthoine

Hauke Busch

Rainer Böckmann

Carlos Camacho

Alessandra Carbone

Robert Castelo

Rui Chen

Jeong-Hyeon Choi

Andrea Cilibierto

Kevin Cohen

Lachlan Coin

Nigel Collier

Attila Csikasz-Nagy

Antoine Danchin

Thomas Dandekar

Vincent Daubin

Hidde de Jong

Xavier De La Cruz

Renee de Menezes

Renee de Menezes

Dick de Ridder

Jeroen de Ridder

Charlotte Deane

Mauro Delorenzi

Emmanouil Dermitzakis

Christophe Dessimoz

Diego Di Bernardo

Andreas Doncic

Jean-Philippe Doyon

Ivana Drobnjak

Omer Dushek

Martin Ebeling

Oliver Ebenhöh

Richard Edwards

Georg Ehret

Laurent Excoffier

Adam Eyre-Walker

Laurent Falquet

Laurent Falquet

Georgios Fengos

Juan Fernandez Recio

Thilo Figge

Samuel Flores

Paul François

Franca Fraternali

Erwin Frey

Caroline Friedel

Dmitrij Frishman

Holger Froehlich

Toni Gabaldon

Julien Gagneur

Nicolas Galtier

Tomas Gedeon

Florian Geier

Olivier Gevaert

Robert Giegerich

Alejandro Giorgetti

Georgios Gkoutos

Giorgio Gonnella

Gaston Gonnet

Berthold Gottgens

Jerome Goudet

Stephan Grill

Andre Gruning

Nicolas Guex

Jürgen Haas

Stefan Haas

Dieter Heermann

Manuela Helmer-Citterich

Jean-Karim Heriche

Jaap Heringa

Henning Hermjakob

Ryan Hernandez

Carl Herrmann

Ralf Herwig

Des Higgins

Michael Hiller

Robert Hoehndorf

Ivo Hofacker

Daniel Hoffmann

Steve Hoffmann

Liisa Holm

Frank Holstege

Hermann-Georg Holzhütter

Rob Hooft

Simon Hubbard

Wolfgang Huber

Martin Huynen

Thomas Höfer

Mark Ibberson

Dagmar Iber

Francesco Iorio

Christian Iseli

Kevin Janes

Alfonso Jaramillo

Klaus Jung

Igor Jurisica

Olga Kalinina

Olga Kalinina

Warren Kaplan

Guy Karlebach

Kazutaka Katoh

Laurence Kelley

Janet Kelso

Patrick Kemmeren

Philipp Khaitovich

Jin-Dong Kim

Jung-Jae Kim

Philip Kim

Wing Kin Sung

Martin Kircher

Hisanori Kiryu

Thomas Klabunde

Gunnar Klau

Judith Klein-Seetharaman

Carole Knibbe

Heinz Koeppl

Oliver Kohlbacher

Rachel Kolodny

Jan Korbel

Sergei Kosakovsky Pond

Roland Krause

Michael Krauthammer

Andriy Kryshtafovych

Gregory Lefebvre

Matthieu Legendre

Hans-Peter Lehnhof

Thomas Lengauer

Ulf Leser

Christina Leslie

Mingyao Li

Hao Lin

Yao-Cheng Lin

Michal Linial

Frederique Lisacek

Hongfang Liu

Nuria Lopez-Bigas

Philip Lord

Daniel MacArthur

Matthias Machacek

Ulrich Mansmann

Paolo Marcatili

John Marioni

Florian Markowetz

Brian Marsden

Lennart Martens

Marc Marti-Renom

Mark McCarthy

Liam McGuffin

Alice McHardy

Dzianis Menshykau

Michael Meyer Hermann

Anke Meyer-Baese

George Michailidis

Andrey Mironov

Shabaz Mohammed

Carmen Molina-Paris

Stephen Montgomery

Sean Mooney

Yves Moreau

Burkhard Morgenstern

Simon Moxon

Matthieu Muffato

Chris Mungall

Felix Naef

Markus Nebel

Goran Nenadic

Zoran Nikoloski

Michael Nilges

Zemin Ning

Magnus Nordborg

Cedric Notredame

John Novembre

Seán O'Donoghue

Jean-Christophe Olivo-Marin

Christine Orengo

Marko Pagni

Jong Park

Kiran Patil

Sabine Peres

Herve Philippe

David Posada

Jaime Prilusky

Nataša Pržulj

Miguel Angel Pujana

Tal Pupko

Zhaohui Qin

Jörg Rahnenführer

Domenico Raimondo

Sanguthevar Rajasekaran

David Rand

Ben Raphael

Dietrich Rebholz-Schuhman

Knut Reinert

Bernhard Renard

Samuli Ripatti

David Ritchie

Eric Rivals

Mark Robinson

Peter Robinson

Marc Robinson-Rechavi

Stephane Rombauts

Michal Rosen-Zvi

Volker Roth

Jacques Rougemont

Rob Russell

Andrey Rzhetsky

Gunnar Rätsch

Sten Rüdiger

Julio Saez-Rodriguez

Nicolas Salamin

Michael Sammeth

Ivo Sbalzarini

Jörg Schaber

Wolfgang Schamel

Avner Schlesinger

Alexander Schliep

Adrian Schneider

Birgit Schoeberl

Michael Schroeder

Jörg Schultz

Marcel Schulz

Stefan Schulz

Stefan Schuster

Russel Schwartz

Torsten Schwede

Benno Schwikowski

Joachim Selbig

Nigam Shah

Roded Sharan

Noam Shomron

Barry Smith

Berend Snel

Johannes Soeding

Ingolf Sommer

Rainer Spang

Alexandros Stamatakis

Oliver Stegle

Jörg Stelling

Robert Stevens

Jens Stoye

Korbinian Strimmer

Josh Stuart

Joakim Sundnes

Eric Tannier

Fabian Theis

Denis Thieffry

Paul Thomas

Peter Tieleman

Jerzy Tiuryn

Anna Tramontano

Achim Tresch

Ala Trusina

Jerry Tsai

David Umulis

Sandor Vajda

Ilya Vakser

Alfonso Valencia

Pascal Vallotton

Roeland van Ham

Jacques van Helden

Antoine van Kampen

Erik van Nimwegen

Vera van Noort

Ceslovas Venclovas

Jean-Philippe Vert

Martin Vingron

Arndt von Haeseler

Christian von Mering

Gert Vriend

Thomas Walter

John Welch

Lodewyk Wessels

Simon Whelan

Edgar Wingender

Ernst Wit

Shoshana Wodak

Jerome Wojcik

Jana Wolf

Verena Wolf

Haim Wolfson

Stephen Wong

Carolina Wählby

Ioannis Xenarios

Julien Yann Dutheil

Mihaela Zavolan

Daniel Zerbino

Louxin Zhang

Xiuwei Zhang

Ralf Zimmer

Andrei Zinovyev

## PROGRAMME COMMITTEE CO-REVIEWERS

Ander, Christina

Arnold, Roland

Atias, Nir

Ayshwarya, Subramanian

Bao, Jie

Bayerlova, Michaela

Berman, Ben

Bohl, Katrin

Bot, Jan

Boulesteix, Anne-Laure

Busse, Dorothea

Bussotti, Giovanni

Carbonell, Pablo

Castro, Mauro

Chen, Li

Chen, Xin

Chinappi, Mauro

Chua, Watson

Cifuentes, Daniel

Colak, Recep

Corcoran, David

Corpas, Manuel

Corre, Tanguy

Csaba, Gergely

Czeizler, Elena

Daminelli, Simone

De Brevern, Alexandre G.

de Oliveira Martins, Leonardo

Degroeve, Sven

Di Camillo, Barbara

Dinh, Hieu

Drewe, Philipp

Dutilh, Bas E.

Erb, Ionas

Erhard, Florian

Felizzi, Federico

Fengos, Georgios

Fischer, Bernd

Fortney, Kristen

Fraternali, Franca

Friedel, Caroline

Fuchs, Christiane

Fuchs, Mathias

Gambardella, Gennaro

Garijo, Daniel

Gerstung, Moritz

Gevaert, Olivier

Gherardini, Pier Federico

Gomes, Mireille

Gonnella, Giorgio

Gonnet, Gaston

Gonzalez,

Gori, Kevin

Griebel, Thasso

Grün, Bettina

Guillemot, Vincent

Han, Xu

Heath, Simon

Helsens, Kenny

Hersch, Micha

Hill, Jamie

Hoehndorf, Robert

Hoener Zu Siederdissen, Christian

Hohm, Tim

Hu, Jialu

Höfer, Thomas

Isik, Zerrin

Jaenicke, Sebastian

Janjic, Vuk

Jeon, Clare

John, Mathias

Jonnalagadda, Siddhartha

Jurman, Giuseppe

Kahles, Andre

Keasar, Chen

Kelemen, Janos

Kemena, Carsten

Kemmeren, Patrick

Knijnenburg, Theo

Knudsen, Michael

Koes, David

Kolbe, Diana

Korcsmaros, Tamas

Kosloff, Mickey

Kowald, Axel

Kramer, Frank

Krumsiek, Jan

Kutalik, Zoltan

Küffner, Robert

Laenen, Griet

Lamparter, David

Lauria, Mario

Le Crom, Stephane

Lee, Miler

Leha, Andreas

Lensink, Marc

Li, Jie

Li, Yong

Lim, Jing Quan

Lord, Phillip

Lorenz, Ronny

Loza, Elisa

Lu, Xiaowen

Luu, Anh Tuan

Mace, Aurelien

Mahfouz, Ahmed

Mahlab, Shelly

Mahony, Shaun

Mancheron, Alban

Mann, Martin

Mansmann, Ulrich

Marek, Diana

Margelevièius, Mindaugas

Marschall, Tobias

Mazza, Arnon

Meyer, Fernando

Michaut, Magali

Mituyama, Toutai

Müller, Nikola

Müller, Tobias

Nicolae, Marius

Oesper, Layla

Olechnovic, Kliment

Olechnoviè, Kliment

Otto, Christian

Pagliarini, Roberto

Palmeri, Antonio

Parca, Luca

Parks, Sarah

Patil, Kaustubh Raosaheb

Pelossof, Raphael

Pemberton-Ross, Peter

Penn, Osnat

Pfeifer, Nico

Philippe, Nicolas

Piasecka, Barbara

Porzelius, Christine

Prunotto, Andrea

Rajan, Vaibhav

Rappoport, Nadav

Rausch, Tobias

Reimann, Matthias

Richard, Hugues

Risso, Davide

Rose, Dominic

Rueedi, Rico

Sakoparnig, Thomas

Schlesner, Matthias

Schoenhuth, Alexander

Setty, Manu

Shao, Chunxuan

Silberberg, Yael

Skunca, Nives

Solari, Aldo

Soneson, Charlotte

Staiger, Christine

Stevenson, Brian

Sundermann, Linda

Taly, Jean François

Ter Horst, Rob

Thomas, Philippe

Torii, Manabu

Tourinsky, Andrei

Tranchevent, Leon-Charles

Travers, Timothy

Tsai, Ming-Chi

Ulrich, Mansmann

Valentini, Giorgio

Valouev, Anton

Valsesia, Armand

van der Lee, Robin

Vandenbon, Alexis

Vandin, Fabio

von der Heyde, Silvia

Vriend, Gert

Waldispuhl, Jerome

Weber, Sebastian

Weigt, Martin

Weinberg, Zasha

Will, Sebastian

Willrodt, Dirk

Woollard, Peter

Yang, Lun

Yang, Rendong

Yaveroglu, Omer Nebil

Yuan, Shuai

Zakrzewski, Martha

Zhang, Zhaolei

Zhao, Meng

Zhong, Yi

